# Effect of Pepper-Containing Diets on the Diversity and Composition of Gut Microbiome of *Drosophila melanogaster*

**DOI:** 10.3390/ijms21030945

**Published:** 2020-01-31

**Authors:** Marleny Garcia-Lozano, Joshua Haynes, Carlos Lopez-Ortiz, Purushothaman Natarajan, Yadira Peña-Garcia, Padma Nimmakayala, John Stommel, Suresh B. Alaparthi, Cristian Sirbu, Nagamani Balagurusamy, Umesh K. Reddy

**Affiliations:** 1 Gus R. Douglass Institute and Department of Biology, West Virginia State University, Institute, WV 25112-1000, USA; mgarcialozano@wvstateu.edu (M.G.-L.); jhaynes18@wvstateu.edu (J.H.); carlos.ortiz@wvstateu.edu (C.L.-O.); pnatarajan@wvstateu.edu (P.N.); ypenagarcia@wvstateu.edu (Y.P.-G.); padma@wvstateu.edu (P.N.); salaparthi@wvstateu.edu (S.B.A.); 2 USDA, ARS, Genetic Improvement of Fruits and Vegetables Laboratory, Beltsville, MD 20705, USA; john.stommel@usda.gov; 3 Charleston Area Medical Center Health Education and Research Institute, Center for Cancer Research, Charleston, WV 25304, USA; cristian.sirbu@camc.org; 4 Laboratorio de Biorremediación, Facultad de Ciencias Biológicas, Universidad Autónoma de Coahuila, Torreón, Coahuila 27000, Mexico

**Keywords:** *Drosophila* gut microbiome, pepper, phytochemicals, diet, genotype

## Abstract

One of the greatest impacts on the gastrointestinal microbiome is diet because the host and microbiome share the same food source. In addition, the effect of diet can diverge depending on the host genotype. Diets supplemented with phytochemicals found in peppers might cause shifts in the microbiome. Thus, understanding how these interactions occur can reveal potential health implications associated with such changes. This study aims to explore the gut microbiome of different *Drosophila* genetic backgrounds and the effects of dietary pepper treatments on its composition and structure. We analyzed the gut microbiomes of three *Drosophila melanogaster* genetic backgrounds (Canton-S, Oregon-RC, and Berlin-K) reared on control and pepper-containing diets (bell, serrano, and habanero peppers). Results of 16S rRNA gene sequencing revealed that the variability of *Drosophila* gut microbiome can be driven mainly by genetic factors. When the abundance of these communities is considered, pepper-containing diets also appear to have an effect. The most relevant change in microbial composition was the increment of Lactobacillaceae and Acetobacteraceae abundance in the pepper-containing diets in comparison with the controls in Oregon-RC and Berlin-K. Regression analysis demonstrated that this enhancement was associated with the content of phenolic compounds and carotenoids of the peppers utilized in this study; specifically, to the concentration of β-carotene, β-cryptoxanthin, myricetin, quercetin, and apigenin.

## 1. Introduction

Microbial communities found in the gut of animals play important roles in the health of the animal through the breakdown of food for nutrient and energy extraction, production of essential vitamins, and protection against pathogen colonization [1]. However, microbiome composition and stability can differ depending on intrinsic factors of the host, such as age, sex, and genotype and exogenous factors including habitat and diet [2]. The addition of specific compounds to the diet may serve to either favorably alter the gut microbiota by promoting the growth of beneficial bacteria or to counteract the imbalance of microbial communities, both with the aim of supporting a healthy host [3].

Chili peppers represent an important crop worldwide due to the beneficial properties of their phytochemicals including carotenoids, capsaicinoids, phenolic compounds, vitamins C and A, and minerals, such as iron and calcium [4]. These compounds have been associated with the control of obesity, the reduction in the risk for coronary disorders, diabetes, cancer, osteoporosis, and neurodegenerative diseases [5,6,7,8].

*Drosophila melanogaster* harbors a simpler gut microbiome as compared to mammals, consisting of various yeast and few bacterial groups, mainly members of the Acetobacteraceae and Lactobacillales taxa [9]. The reduced complexity of these microbial associations in the gut of *Drosophila* has facilitated the analysis and hypothesis testing of the microbiota–host interactions on the nutritional phenotype of the host [10]. Nevertheless, bacterial communities can be altered by different parameters, which in turn shape these microbiota–host interactions [11]. Several studies have found that diet plays a crucial role in the shifting of bacterial groups of *Drosophila* [12,13,14]. In addition, host-genotype specific factors also have been observed to shape the gut microbiome of *D. melanogaster* [15,16].

The main objective of this study is to explore the changes in composition and diversity of the gut microbiota of different *Drosophila* genetic backgrounds reared on diets supplemented with bell, serrano, and habanero peppers. Moreover, this study aims to reveal microbial–microbial interactions between members of the *Drosophila* microbiome. 

## 2. Results

### 2.1. Phytochemical Content

We estimated the content of flavonoids, carotenoids, and capsaicinoids in the peppers utilized to supplement the different diets (Table 1). It was observed that almost all the compounds were detected in the different peppers, although the concentration varied. Bell pepper contained the highest concentration of β-carotene (129.153 µg/g), capsanthin, (52.001 µg/g) and the flavonoid quercetin (41.355 µg/g) and no capsaicinoid content was detected in this pepper, whereas the highest concentration of capsaicin (2529.117 µg/g) and dihydrocapsaicin (1524.960 µg/g) was detected in serrano pepper. In habanero pepper, the highest concentration of the flavonoid apigenin was reported, but in general, this pepper contained the lowest concentration of carotenoids and flavonoids, although it had a significant content of capsaicinoids.

### 2.2. Sequencing Data

The total number of sequences obtained from the 16S rRNA gene amplicons resulting from Illumina sequencing was 22,780,501 (range 672,415 to 1,392,635 per sample). After filtering and de-noising by using DADA2, 3,788,511 clean and non-chimeric reads were obtained and ASVs were generated for further taxonomic classification. ASVs belonging to the endosymbiont *Wolbachia*, mitochondria, and chloroplasts were removed, and a total of 409 ASVs were denominated as the *Drosophila* microbiome, with an average of 50 ± 13 ASVs per sample.

### 2.3. Gut Microbiome Structure of D. melanogaster Strains Reared on the Different Diets

We calculated alpha and beta diversity metrics to investigate whether the structure of the gut microbiota of different wild-type *Drosophila* strains diverged and if these communities were responding to the pepper-containing diets. Alpha diversity analysis was utilized to calculate species diversity in a sample through different indices, including observed species (richness), Chao1, and Shannon and Simpson. Results of alpha diversity analysis showed that the richness of gut microbial communities differed between the genetic backgrounds. Species richness was higher in Canton-S (*p* = 0.001) and Oregon-RC (*p* = 0.001) compared with Berlin-K. Additionally, microbial communities had greater richness in Canton-S than Oregon-RC (*p* = 0.001) (Figure S1, Tables S1 and S2). 

**Figure 1:**
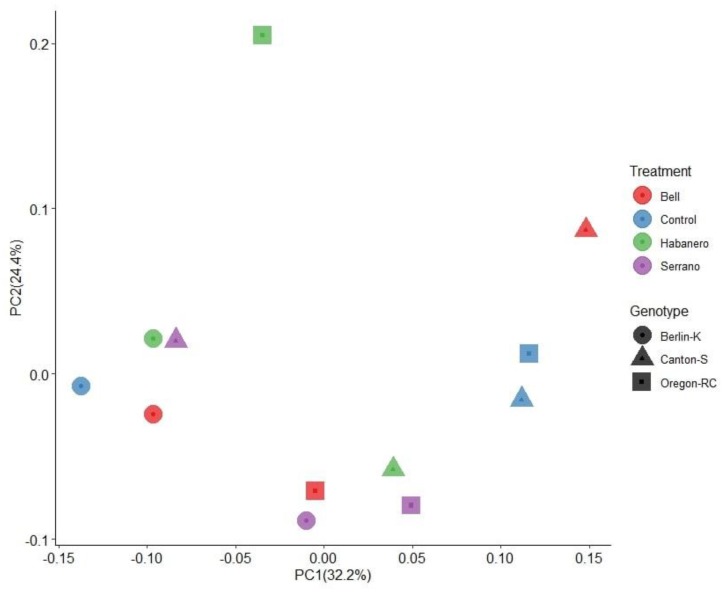
Principal coordinate analyses (PCoA) plot based on unweighted UniFrac distance between genetic backgrounds and diets. This figure shows the percentage of variation explained by the first two principal components (P1, P2). Each symbol represents the average of two biological replicates.

Beta diversity was calculated to determine differences in the composition and abundance of microbial groups among the samples. Beta diversity was calculated using UniFrac distances, which compare the phylogenetic distances of the microbial groups. These comparisons were made based on microbial abundance and presence/absence data by using weighted and unweighted UniFrac indices, respectively. The variation in the unweighted UniFrac PCoA plot was explained by PC1 (38.5%) and PC2 (25.6%), accounting for a total of 64.1.3% of the overall diversity between the samples (Figure 1). Based on this analysis, microbiome composition varied significantly between host genetic backgrounds but was not perturbed by the pepper-containing diets (PERMANOVA, *p* = 0.002, 1000 permutations) (Table S3), as can be observed in the clustering pattern of the different genetic backgrounds in Figure 1.

**Figure 2:**
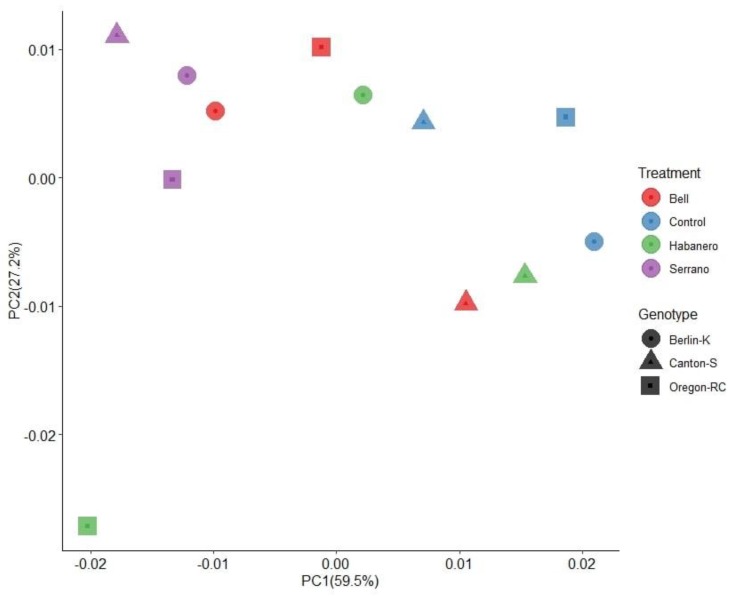
Principal coordinate analyses (PCoA) plot based on weighted UniFrac distances between genetic backgrounds and diets. This figure shows the percentage variation explained with the first two principal components (P1, P2). Each symbol represents the average of two biological replicates.

Pairwise comparisons of unweighted UniFrac distances revealed significantly different gut microbiomes between Berlin-K and Canton-S (*p* = 0.003). Additionally, gut microbial communities of Oregon-RC statistically differed from those of Berlin-K (*p* = 0.01) (Table S4). However, when the abundance of ASVs was considered (weighted UniFrac distance), the diets appeared to influence the microbiome structure of the *Drosophila* genetic backgrounds (PERMANOVA, *p* < 0.01, 1000 permutations) (Table S5). Additionally, the PCoA plot for this distance explained a greater amount of variation (89.2% of overall diversity; Figure 2). Pairwise comparisons revealed that microbial communities in *Drosophila* genetic backgrounds reared on the serrano-containing diet significantly differ from those detected in the control diet (*p* = 0.042; Table S6). 

### 2.4. Gut Microbiota Composition of Drosophila Genetic Backgrounds Reared on the Different Diets

**Figure 3:**
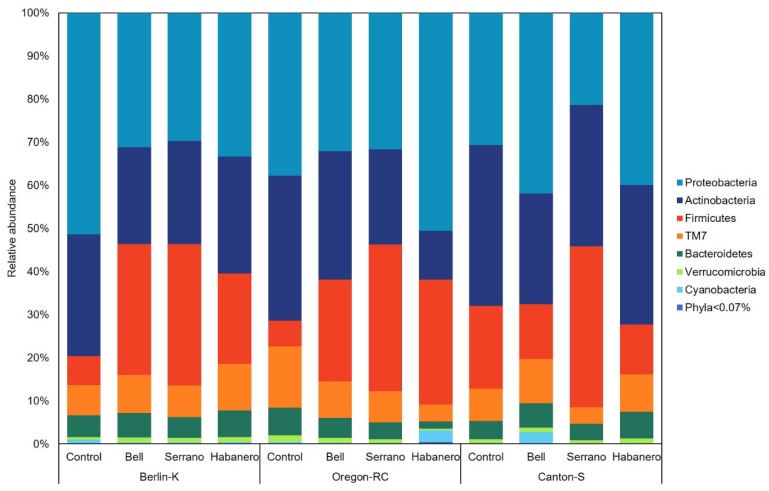
Relative abundance of different phyla in the *Drosophila* genetic backgrounds reared on the different diets.

To further determine the association between the genetic backgrounds and pepper-containing diets in the gut microbiota composition, a taxonomy-based analysis was performed at different levels. Specific microbial taxa differed in abundance depending on the diet. The most abundant phylum among all samples was Proteobacteria (35.98%), followed by Actinobacteria (27.23%), Firmicutes (21.99%), TM7 (8.2%), and Bacteroidetes (4.86%) (Figure 3).

Proteobacteria represented more than 50% of the microbiome in Berlin-K flies reared on the control diet, although this phylum was found in less abundance in flies raised on pepper-containing diets (29.78–33.37%). In contrast, Firmicutes was found in much greater abundance in these gut microbiomes (21–32.79%) as compared with microbial populations of flies maintained on control diet (6.72%). This phenomenon was also observed in gut microbial communities of Oregon-RC flies, where Firmicutes abundance increased from 6.06% in flies with the control diet to 23.55 to 34.04% in flies with pepper-containing diets. Conversely, Actinobacteria proportion decreased from 33.58% with the control diet to 22.05 and 11.36% with serrano- and habanero-containing diets, respectively. 

**Figure 4:**
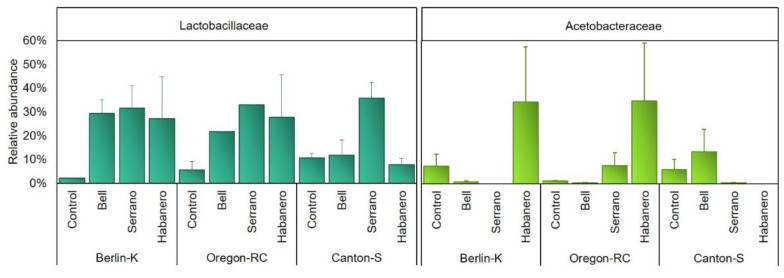
Relative abundance of Lactobacillaceae and Acetobacteraceae families in the *Drosophila* genetic backgrounds reared on the different diets.

In this study, the microbiome at the family level showed Lactobacillaceae (30.11%) and Acetobacteraceae (10.67%) among the most abundant families across pepper-containing diets (Figure S2). Berlin-K and Oregon-RC flies reared on habanero-containing diets showed a high abundance of both microbial groups. In addition, Lactobacillaceae was found in greater abundance in Berlin-K and Oregon-RC flies raised on all pepper-containing diets as compared with flies maintained on the control diet (Figure 4). Lactobacillaceae was dominated by genera within the order Lactobacillales (*Pediococcus* and *Lactobacillus*). *Lactobacillus* was mainly represented by *Lactobacillus brevis* (Figure S3)*. L. brevis* was greatly abundant in Berlin-K flies reared on bell pepper-containing diet, whereas in Oregon-RC flies, *L. brevis* abundance was 4-fold higher in flies reared on pepper-containing diets than on the control diet. 

Members of the Acetobacteraceae family were represented mainly by the *Acetobacter* genus, which exhibited variable abundance (Figure S4). Oregon-RC and Canton-S flies raised on pepper-containing diets showed a higher abundance of this genus as compared with flies maintained on control diets. Actinobacteria, one of the main phyla present in this study, accounted for more than 27% of the gut microbiomes. This phylum was more abundant across all genetic backgrounds reared on the control diet (28.27–37.33%) than pepper-containing diets (11.36–32.75%).

**Figure 5:**
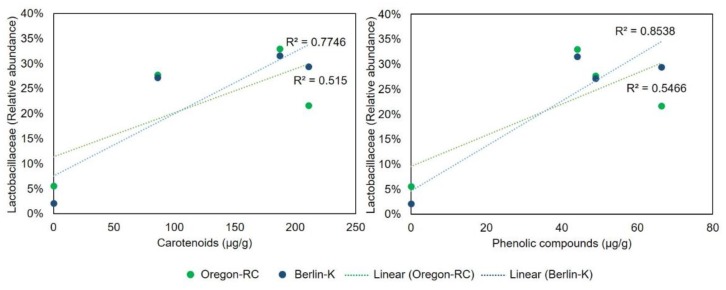
Relationship between Lactobacillaceae and Acetobacteraceae abundance in Oregon-RC and Berlin-K flies and the concentration of various compounds.

**Figure 6:**
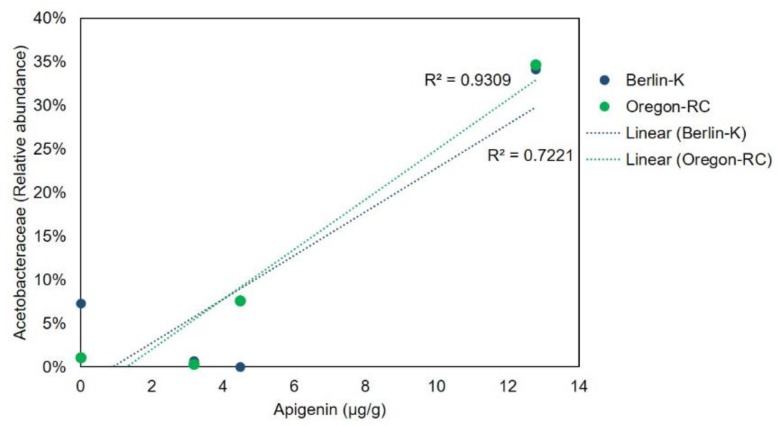
Relationship between Acetobacteraceae abundance in Oregon-RC and Berlin-K flies and apigenin concentration in the dietary treatments.

The effect of the pepper-containing diets on the Lactobacillaceae and Acetobacteraceae abundance was supported by the regression analysis presented in Figure 5 and Figure 6. These graphs show the correlation between Lactobacillaceae and Acetobacteraceae abundances and the concentration of various compounds. Lactobacillaceae abundance in Berlin-K and Oregon-RC flies was positively correlated to the content of phenolic compounds and carotenoids. As these compounds increased in the pepper diets, the abundance of Lactobacillaceae was also found to be augmented. Furthermore, Acetobacteraceae abundance showed a positive correlation with the concentration of the phenolic compound apigenin in these two genetic backgrounds (Figure 6). As habanero peppers contained a considerable concentration of apigenin (Table 1), the great abundance of Acetobacteraceae in Berlin-K and Oregon-RC flies reared on habanero-containing diets may be attributed to this compound. 

**Figure 7:**
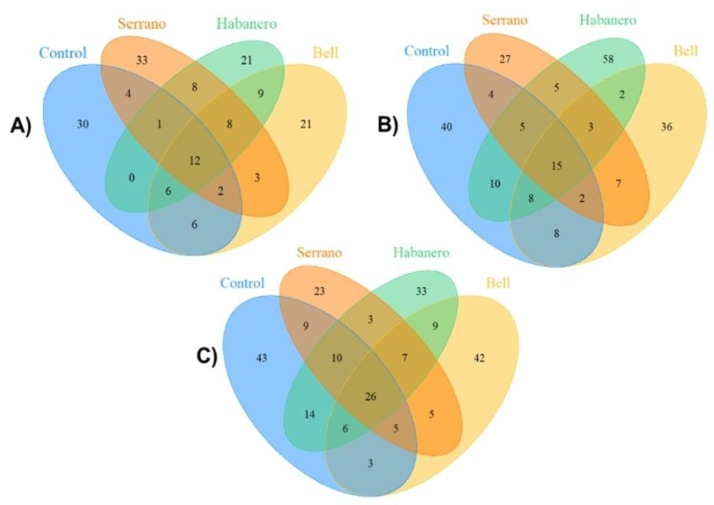
Venn diagrams showing the number of unique and shared ASVs between the *Drosophila* genetic backgrounds under different diets. (**A**) Berlin-K, (**B**) Oregon-RC and (**C**) Canton-S flies.

The core microbiome in the different genetic backgrounds is represented in Figure 7. In Berlin-K flies, 12 ASVs were consistently present with all diets. They belonged mainly to Alphaproteobacteria, Bacteroidetes, and Actinomycetales classes, but 15 ASVs belonging to Actinobacteria, Alphaproteobacteria, Gammaproteobacteria, and Flavobacteria were common in Oregon-RC flies under all diets. In addition, 26 ASVs belonging to Actinobacteria, Alphaproteobacteria, Bacilli, and Flavobacteria were present in Canton-S flies. Moreover, their abundance strikes as being mediated by the dietary pepper treatments.

### 2.5. Microbiome Interaction Networks among Drosophila Populations Reared on Different Diets

**Figure 8:**
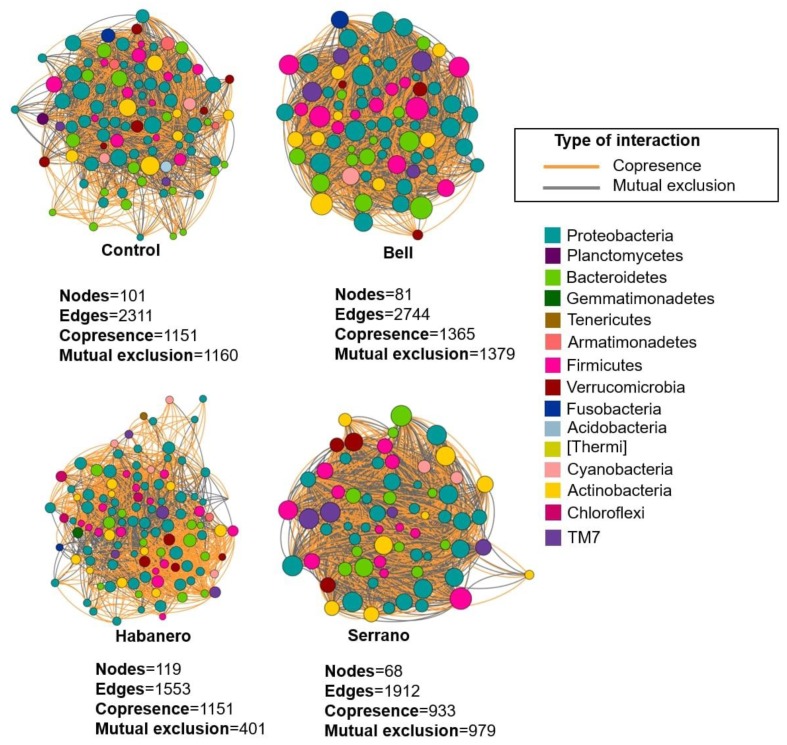
Microbial interaction networks of Oregon-RC under the different diets. Node colors represent taxonomy identifiers of the ASVs at the phylum level. Orange edges represent co-presence/positive correlation and gray edges represent mutual exclusion/negative correlation. Multiple edges connecting the same nodes indicate significance for more than one metric (Bray–Curtis dissimilarity, Kullback–Leibler divergence, mutual information, Spearman and Pearson correlations). Node size is proportional to the outcoming connected edges (outdegree).

As we identify that the Oregon-RC microbiome clearly differed in the pepper-containing diets, as compared with the control diet, interaction networks were inferred from bacterial ASVs of this genotype (Figure 8). Distinct co-occurrence patterns were generated across the diets. For all the diets, we observed both co-occurrence and co-exclusion interaction patterns in all networks. In general, these networks revealed more negative than positive co-associations. Overall, the most abundant phyla, including Proteobacteria, Actinobacteria, Firmicutes, and TM7, showed different co-association patterns across the diets. We identify the hubs of these networks by considering the highly connected nodes positively or negatively affecting other neighbor nodes (max outdegree).

The identified taxa within these interactions that appeared to be important to the overall structure of the networks were Proteobacteria, Firmicutes, Bacteroidetes, Actinobacteria, TM7, and Verrucomicrobia. In the control diet, ASVs belonging mainly to Actinomycetales (Actinobacteria), Pseudomonadales (Gammproteobacteria), and Rhizobiales (Alphaproteobacteria) were the most important nodes in the network. Whereas in pepper-containing diets, ASVs belonging to Lactobacillales (Bacilli), Bacillales (Bacilli), Flavobacteriales (Flavobacteriia), Procabacteriales (Betaproteobacteria), and I025 (TM7-3) had the highest outdegree. Additionally, in bell-pepper containing diets, the species *L. brevis*, *Bacillus clausii*, and *Flavobacterium columnare* were the hubs of the network.

## 3. Discussion

In this study, we investigated the gut microbiota of different *Drosophila* genetic backgrounds and whether the composition and structure of these microbiomes are affected by different pepper containing diets. Alpha and beta diversity analyses revealed that *Drosophila* microbiome composition was influenced primarily by genotype than diet. These results agree with a genome-wide association study showing that about 78% of the variance of microbial community composition was attributed to the host genotype [17]. Oregon-RC and Canton-S having greater richness than Berlin-K but similar diversity suggests that Oregon-RC and Canton-S had a large number of different ASVs than Berlin-K, but those were at low abundance.

Pepper-containing diets also appeared to influence gut microbiome composition of the different *Drosophila* genetic backgrounds based on PERMANOVA of weighted UniFrac distance, and this phenomenon was observed on the variability of the relative abundance of the different taxa. At the phylum level, the microbial populations among all *Drosophila* genetic backgrounds were principally composed by Proteobacteria, Actinobacteria, Firmicutes, and TM7. Except for TM7, these phyla have also been reported in previous studies of the *Drosophila* microbiome [18].

Species belonging to Proteobacteria and Firmicutes are important members of *Drosophila* microbiota [19]. Studies of laboratory and wild *Drosophila* have reported that its microbiota mainly contains families belonging to these phyla, including Lactobacillaceae, Enterococcaceae, Acetobacteraceae, and Enterobacteriaceae. In addition, microbial groups belonging to Lactobacillus and Acetobacter genera are the most common [20,21]. Nonetheless, these microbial groups were barely abundant in the control diets, where it was expected to observe the standard *Drosophila* microbiome. In Oregon-RC and Berlin-K genotypes, the most abundant groups in control diets belonged to unclassified members of Actinomycetales, Rhizobiales, and Proteobacteria (Figure S2). Thus, it is evident that there was an observable change in the *Drosophila* microbiome in the diets spiked with peppers in comparison to the controls in this study. More specifically, pepper-containing diets appeared to enhance members of Lactobacillaceae and Acetobacteraceae in *Drosophila* gut microbiome when compared to the controls. Regression analysis showed the positive correlation between the abundance of these bacterial families and the content the pepper compounds utilized in this study. While Lactobacillaceae appeared to be positively correlated with the concentration of phenolic compounds and carotenoids in Oregon-RC and Berlin-K flies, Acetobacteraceae was positively correlated to the content of the phenolic compound apigenin.

The abundance of *L. brevis* belonging to Lactobacillaceae was 4-fold higher in flies reared on pepper-containing diets than in guts of flies raised on the control diet. In addition, this species was also greatly abundant in flies fed with bell pepper-containing diets of Berlin-K genotype. As all peppers contained similar levels of the carotenoids β-carotene and β-cryptoxanthin, and of the phenolic compounds myricetin and quercetin, we hypothesize that these compounds may be involved in the promotion of *L. brevis* abundance.

Networks of the Oregon-RC genotype fed with pepper diets showed Rhizobiales, Actinomycetales, and Pseudomonadales were not hubs of these networks as they were in control diets, indicating the pepper diets caused a perceptible shift in networking (Figure 8). Furthermore, network structure was altered under different pepper-containing diets, serrano and bell peppers being the main shapers of the microbial community.

## 4. Materials and Methods

### 4.1. Drosophila Stocks and Cultures

Three classical wild-type strains of Drosophila melanogaster, including Oregon-RC (5), Canton-S (64349), and Berlin-K (8522), were obtained from the Bloomington Stock Centre (Indiana University, Bloomington, Indiana). All flies were originally maintained on a standard sugar-yeast agar media.

### 4.2. Maintenance of Drosophila Strains on Control and Pepper Containing Diets

Populations of all three genetic backgrounds were placed onto four different diets: control and bell pepper-, habanero-, and serrano-containing diets. The pepper-containing diets consisted of autoclave-sterilized standard cornmeal medium (Nutri-fly Bloomington formulation, Genesee Scientific, San Diego, CA, USA) solidified with agar and supplemented with 0.4% propionic acid (*v/v*) and 0.3% Tegosept (*w/v*) as preservatives. In addition, the diets contained 2% (*w/w*) ground and dried peppers including habanero, serrano, and bell pepper. The control diet included this formulation except for pepper supplementation. All experiments and culturing were performed in controlled conditions at 25 °C on a 12 h light/dark photoperiod. Experiments were initiated by placing approximately 10 males and 10 females of each genotype onto vials containing the different diets. Adults were allowed to lay eggs for 72 h before being removed. The larvae were fed and once the adult stage was achieved, these flies were selected for gut dissection. Each of the control and pepper containing lines were maintained in three independent lines of vial culture. 

### 4.3. Gut Dissection and DNA Extraction

For DNA extraction, guts were dissected by using a modified protocol described by [22] and [16]. Twenty flies were selected for each sample and were surface sterilized with 5% sodium hypochlorite. Guts were dissected under a dissecting microscope, with flies placed in sterile Ringer’s solution on sterile Petri dishes and the use of sterilized forceps. The dissected midguts were placed into a 2-mL tube for each sample. An approximately equal number of males and females from each treatment were dissected to limit potential variability based on sex.

The dissected gut tissue was homogenized by grinding with plastic pestles inside 2-mL microcentrifuge tubes and three freeze/thaw cycles in liquid nitrogen. A 180-µL amount of lysis buffer (20 mM tris-HCl pH 8.0, 2 mM sodium EDTA, 1.2% Triton-X 100, with 20 mg/mL freshly added lysozyme) was added and samples were incubated at 37 °C for 90 min, with vortexing at maximum speed for 3 min after 45 min. A 20 µL amount of extraction buffer (2 M Tris-HCl pH 8.0, 2.5 M NaCl, 0.25 M EDTA, 5% *w/v* SDS buffer) and 15 µL proteinase K were added to each sample and samples were further incubated for 30–60 min at 55 °C. Samples were precipitated by incubation at room temperature for 30 min in 30 µL of 3 M sodium acetate, inverting the tubes every 10 min. The samples were centrifuged at 11,000× *g* for 10 min and the supernatant was mixed with 300 µL of 100% ice-cold isopropanol and incubated at room temperature for 30 min, followed by centrifuging at 18,000× *g* for 30 min. The supernatant was discarded, and the pellets were washed in 70% ice-cold EtOH, air dried, and resuspended in 20 µL of 10 mM Tris-HCl, pH 8.5, for elution.

### 4.4. PCR Conditions and Product Purification

For amplification of the 16S rRNA gene, bacterial universal primers 341F (5′-CCTAYGGGRBGCASCAG-3′) and 806R (5′-GGACTACNNGGGTATCTAAT-3′) were used to amplify the V3–V4 variable regions. PCR involved a modified PCR protocol from [23]: initial denaturation at 94 °C for 3 min followed by 45 cycles of 94 °C for 45 sec, 50 °C for 60 sec, and 72 °C for 90 sec, with a final extension at 72 °C for 10 min. Products were checked by 1% gel electrophoresis and purified by using the Qiagen QiaQuick PCR purification kit (Germantown, MD, USA).

### 4.5. Library Preparation and Sequencing

Sequencing libraries were generated by using the NEB Next^®^ Ultra™ DNA Library prep kit for Illumina (NEB, Ipswich, MA, USA) following the manufacturer’s recommendations. Indexed libraries were quantified with a Qubit HS DNA kit on a Qubit Fluorometer and the quality was assessed by using an Agilent Bioanalyzer 2100 system (Santa Clara, CA, USA). After normalization and pooling, libraries were sequenced on an Illumina NextSeq 500 platform, generating 150 paired-end reads. Demultiplexing of indexed reads and generation of FASTQ files were performed using Illumina’s bcl2fastq Conversion software v.1.8.4. Sequences were deposited in the NCBI SRA repository under BioProject ID: PRJNA556953 (accession numbers SAMN12385229-SAMN12385252).

### 4.6. Data Analysis

The sequence quality of raw reads was first assessed by using FastQC [24]. Trim Galore software version 0.50 was used for quality and adapter trimming of the raw reads. Clean and demultiplexed reads were then imported and processed by using QIIME2 software version 2018.8 [25]. DADA2 pipeline was used for quality control and chimera removal. This method allows for correcting Illumina amplicon errors without the construction of operational taxonomic units. In contrast, DADA2 infers amplicon sequence variants (ASVs), which represent real biological sequences at single nucleotide resolution [26]. ASVs were resolved only from forward reads and were further taxonomically assigned by using a pre-trained classifier, Greengenes, for 99% of the V3–V4 16S gene region. ASVs belonging to the endosymbiont *Wolbachia,* mitochondria and chloroplasts were filtered from the data. For constructing taxa plots, replicates were merged, which resulted in a total of 12 groups, including 3 genetic backgrounds subjected to 4 different diets. The output ASV table was exported from QIIME2 and used as input in R for downstream analysis. Shared and unique taxa among the different genetic backgrounds and diets were identified by using the package VennDiagram v1.6.20 in R [27] to reveal the core gut microbiome in *Drosophila* reared on the different diets.

### 4.7. Diversity Metrics

Alpha and beta diversity metrics were used to estimate the microbial community diversity across all samples. Alpha diversity was calculated by using the phyloseq package version 1.30.0 [28] according to metrics, including observed species (richness), expected species (Chao1), the Simpson dominance index, and Shannon equality index. Alpha diversity metrics among genetic backgrounds and diets were compared using two-way-analysis of variance (ANOVA) with the Tukey–Kramer post hoc test to analyze differences between treatments and genetic backgrounds.

Beta diversity was determined by calculating weighted and unweighted UniFrac distance matrices, which were further visualized by principal coordinates analysis (PCoA). The Adonis function of the Vegan package version 2.4-4 in R [29,30] was used for permutational multivariate analysis of variance (PERMANOVA) of both weighted and unweighted UniFrac distance matrices. Pairwise PERMANOVA comparisons were carried out with the package pairwiseAdonis version 0.3 [31] in R. 

### 4.8. Network Analysis

Co-occurrence networks were constructed to identify the interactions between ASVs by using the feature table from QIIME. Networks were created by using CoNet [32] according to the following methodology: All taxa below a minimum occurrence of 20 across the samples were deleted, and the counts were converted into relative abundance. Thereafter, a distribution of all pairwise scores was computed for each of five similarity measures including Bray–Curtis dissimilarity, Kullback–Leibler divergence, mutual information, and Spearman and Pearson correlations. Given these distributions, initial thresholds were selected for the initial network, which had 1000 positive and 1000 negative edges supported by all five measures. *P*-values were computed from method- and edge-specific permutation and bootstrap score distributions with 1000 iterations each. Measure-specific *p*-values were merged by using Brown’s method; Benjamini–Hochberg’s false discovery rate correction was applied and edges with merged *p* < 0.5 were retained. Co-occurrence networks were visualized by using Cytoscape v3.7.1 [33] with the implemented organic layout. The resulting statistically significant interactions were classified as co-presence, mutual exclusion, or unknown. Because unknown interactions cannot describe an interaction pattern, they were excluded from the networks. Statistical analyses involved using Cytoscape with NetworkAnalyzer [34].

### 4.9. Quantification of Phytochemicals

Flavonoid, carotenoid and capsaicinoid content of the peppers utilized in this study were estimated by the following methodology: Flavonoid content was quantified as described by Bae et al. [35] with some modifications. One gram of fresh pepper was homogenized with 10 mL of methanol for 2 min and extracted on vortex for 15 min at room temperature. Two milliliters of extract solution were treated with 3 mL of 3 M HCl at 75 °C in a water bath for 1 h. The hydrolyzed sample was cooled to room temperature and filtered through a Phenomenex 0.2 μm PTFE membrane filter (Torrance, CA, USA) before analysis. The sample was further transferred into pre-labeled HPLC auto sampler vials and 10.0 µL was injected for HPLC analysis. The mobile phases were methanol and 2% orthophosphoric acid (*v/v*) at a flow rate of 0.6 mL/min. An X-Bridge C18 column (4.6 × 150 mm; 5 μm) coupled with a guard column (Waters Corp.) was used. The flavonoid compounds levels were detected at 360 nm. Stock solutions of flavonoids (Sigma-Aldrich, St. Louis, MO, USA) were prepared in methanol for a linear standard curve ranging from 0.25 to 12.5 µg/mL.

Carotenoid compounds were estimated as described by Chebrolu et al. [36] and Yoo et al. [37] with modifications. One gram of fresh pepper was homogenized for 2 min with 10 mL of acetone and 0.1% BHT (butylated hydroxytoluene) and extracted on vortex for 15 min at room temperature. Six milliliters of extract solution were mixed with 10 mL of hexane and 15 mL of water to further separate the hexane layer from the acetone/water layer. The hexane extract was freeze dried under nitrogen gas with a Reacti-Vap Evaporator system (Pierce Biotech, Rockford, IL, USA). Then, the sample was reconstituted with 1 mL of acetone and an aliquot of 10.0 µL of the final solution was injected for HPLC analysis. The mobile phases were 0.1% BHT MTBE and acetone (*v/v*) at a flow rate of 0.8 mL/min. An YMC Carotenoid C30 column (4.6 × 250 mm; 3 μm) coupled with a guard column (Waters Corp) was used. The carotenoids compounds levels were detected at 450 nm. Stock solutions of carotenoids (Sigma-Aldrich) were prepared in acetone for a linear standard curve ranging from 0.5 to 25.0 µg/mL. The pepper sample of capsaicin and dihydrocapsaicin content was performed as described by Nimmakayala et al. [38]. The HPLC system was equipped with a 1525 binary HPLC pump, 2707 autosampler, and 2998 photodiode array detector (Waters Corp., Milford, MA, USA).

## 5. Conclusions

Here, we presented an exploratory analysis of *Drosophila* gut microbiome and the effects of pepper-containing diets on its structure, networking, and composition. This study revealed that *Drosophila* gut microbiome can be shifted by pepper-containing diets, but the response varied according to the genotype and pepper type. Restoration or promotion of standard members of the gut microbiome appeared to be mediated by the pepper diet. This effect might be attributed to the various metabolites of the peppers, including β-carotene, β-cryptoxanthin, myricetin, quercetin, and apigenin, though at present we do not know the individual effect of these compounds on fly physiology and health. Based on this information, focused studies involving more diverse individual genetic backgrounds can be performed using the purified compounds.

## Figures and Tables

**Table 1:** Content of the main phytochemical compounds in the different peppers utilized in this study.

	Bell	Serrano	Habanero
Carotenoids (µg/g)			
Capsanthin	52.001	37.676	11.145
α-carotene	12.88	23.435	1.781
β-carotene	129.153	115.670	64.470
β-cryptoxanthin	16.845	10.267	8.816
Phenolic compounds (µg/g)			
Myricetin	9.672	8.864	4.979
Quercetin	41.355	26.028	21.532
Kaempferol	ND	ND	0.801
Apigenin	3.177	4.483	12.782
Luteolin	12.140	4.672	8.764
Capsaicinoids (µg/g)			
Capsaicin	ND	2529.117	2478.723
Dihydrocapsaicin	ND	1524.960	746.127

## Supplementary Materials

The following are available online at https://www.mdpi.com/1422-0067/21/3/945/s1.
