# Supplementary material for: Effect of Pepper-Containing Diets on the Diversity and Composition of Gut Microbiome of Drosophila melanogaster
Source: Int J Mol Sci. 2020 Jan 31;21(3):945. doi: 10.3390/ijms21030945 (PMC7038135; doi:10.3390/ijms21030945)
Supplement: Supplementary file 1 [file ijms-21-00945-s001.zip › ijms-670590-SI/Figure Captions.docx]

Figure S1: Alpha diversity metrics among all the genetic backgrounds and treatments;

Figure S2: Relative abundance in gut microbiota at family level among *Drosophila* genetic backgrounds and diets;

Figure S3: Relative abundance in gut microbiota at species level among *Drosophila* genetic backgrounds and diets;

Figure S4: Relative abundance in gut microbiota at genus level among *Drosophila* genetic backgrounds and diets;

Table S1: Results of two-way ANOVA to compare richness between genetic backgrounds and treatments;

Table S2: Results of Tukey–Kramer pos hoc test for richness values calculated across the *Drosophila* genetic backgrounds;

Table S3: Results of PERMANOVA analysis of gut microbiome composition between *Drosophila* genetic backgrounds maintained on different diets based on unweighted UniFrac distances;

Table S4: Results of pairwise comparisons of *Drosophila* genetic backgrounds under different diets;

Table S5: Results of PERMANOVA analysis of gut microbiome composition between *Drosophila* genetic backgrounds maintained on different diets based on weighted UniFrac distances;

Table S6: Results of pairwise comparisons of dietary treatments across *Drosophila* genetic backgrounds.
