# Supplementary material for: Effect of Pepper-Containing Diets on the Diversity and Composition of Gut Microbiome of Drosophila melanogaster
Source: Int J Mol Sci. 2020 Jan 31;21(3):945. doi: 10.3390/ijms21030945 (PMC7038135; doi:10.3390/ijms21030945)
Supplement: Supplementary file 1 [file ijms-21-00945-s001.zip › ijms-670590-SI/Table S1.docx]

**Table S1.** Results of two-way ANOVA to compare richness between genetic backgrounds and treatments.

| Variable | df | Sum of Squares | Mean of Squares | F value | Pr(>F) |
| --- | --- | --- | --- | --- | --- |
| Treatment  Genotype  Treatment: Genotype  Residuals | 3  2  6  12 | 351.2  2308.1  688.6  320 | 117.1  1154  114.8  26.7 | 4.39  43.277  4.304 | 0.0265  3.26E-06  0.0152 |
