# Supplementary material for: Effect of Pepper-Containing Diets on the Diversity and Composition of Gut Microbiome of Drosophila melanogaster
Source: Int J Mol Sci. 2020 Jan 31;21(3):945. doi: 10.3390/ijms21030945 (PMC7038135; doi:10.3390/ijms21030945)
Supplement: Supplementary file 1 [file ijms-21-00945-s001.zip › ijms-670590-SI/Table S2.docx]

| Comparison | difference | lower | upper | p adj |
| --- | --- | --- | --- | --- |
| Berlin-K to Canton-S | 24 | 17.111606 | 30.888394 | 0.0000022 |
| Berlin-K to Oregon-RC | 12.875 | 5.986606 | 19.763394 | 0.0008531 |
| Canton-S to Oregon-RC | 11.125 | -18.013394 | -4.236606 | 0.0026983 |

**Table S2.** Results of Tukey-Kramer pos hoc test for richness values calculated across the *Drosophila* genetic backgrounds.
