# Supplementary material for: Effect of Pepper-Containing Diets on the Diversity and Composition of Gut Microbiome of Drosophila melanogaster
Source: Int J Mol Sci. 2020 Jan 31;21(3):945. doi: 10.3390/ijms21030945 (PMC7038135; doi:10.3390/ijms21030945)
Supplement: Supplementary file 1 [file ijms-21-00945-s001.zip › ijms-670590-SI/Table S3.docx]

**Table S3.** Results of PERMANOVA analysis of gut microbiome composition between each of the genetic backgrounds lines maintained on the different diets based on unweighted UniFrac distances.

| Variable | df | Sums of squares | Mean squares | F | R^2^ | P |
| --- | --- | --- | --- | --- | --- | --- |
| Treatment | 3 | 0.0698 | 0.0232 | 1.012 | 0.1042 | 0.456 |
| Genotype | 2 | 0.1387 | 0.0693 | 3.017 | 0.2070 | 0.002 |
| Treatment:Genotype | 6 | 0.1856 | 0.0309 | 1.345 | 0.2769 | 0.123 |
| Residuals | 12 | 0.2759 | 0.0229 |  | 0.4117 |  |
| Total | 23 | 0.6701 |  |  | 1 |  |
