# Supplementary material for: Effect of Pepper-Containing Diets on the Diversity and Composition of Gut Microbiome of Drosophila melanogaster
Source: Int J Mol Sci. 2020 Jan 31;21(3):945. doi: 10.3390/ijms21030945 (PMC7038135; doi:10.3390/ijms21030945)
Supplement: Supplementary file 1 [file ijms-21-00945-s001.zip › ijms-670590-SI/Table S4.docx]

**Table S4.** Results of pairwise comparisons of *Drosophila* genetic backgrounds reared on the different diets.

| Pairs | df | Sums of squares | F.Model | R^2^ | p.value | p.adjusted |
| --- | --- | --- | --- | --- | --- | --- |
| Oregon-RC vs Berlin-K | 1 | 0.0714 | 2.7822 | 0.1657 | 0.01 | 0.03 |
| Oregon-RC vs Canton-S | 1 | 0.0432 | 1.4632 | 0.0946 | 0.132 | 0.396 |
| Berlin-K vs Canton-S | 1 | 0.0934 | 4.5236 | 0.2442 | 0.001 | 0.003 |
