# Supplementary material for: Effect of Pepper-Containing Diets on the Diversity and Composition of Gut Microbiome of Drosophila melanogaster
Source: Int J Mol Sci. 2020 Jan 31;21(3):945. doi: 10.3390/ijms21030945 (PMC7038135; doi:10.3390/ijms21030945)
Supplement: Supplementary file 1 [file ijms-21-00945-s001.zip › ijms-670590-SI/Table S5.docx]

**Table S5.** Results of PERMANOVA analysis of gut microbiome composition between each of the genetic backgrounds lines maintained on the different diets based on weighted UniFrac distances.

| Variable | df | Sums of squares | Mean squares | F | R^2^ | P |
| --- | --- | --- | --- | --- | --- | --- |
| Treatment | 3 | 0.1093 | 0.0364 | 2.0809 | 0.2728 | 0.077 |
| Genotype | 2 | 0.0141 | 0.0070 | 0.4031 | 0.0352 | 0.846 |
| Treatment:Genotype | 6 | 0.0670 | 0.0111 | 0.6379 | 0.1673 | 0.816 |
| Residuals | 12 | 0.2101 | 0.0175 |  | 0.5245 |  |
| Total | 23 | 0.4006 |  |  | 1 |  |
