# Supplementary material for: Effect of Pepper-Containing Diets on the Diversity and Composition of Gut Microbiome of Drosophila melanogaster
Source: Int J Mol Sci. 2020 Jan 31;21(3):945. doi: 10.3390/ijms21030945 (PMC7038135; doi:10.3390/ijms21030945)
Supplement: Supplementary file 1 [file ijms-21-00945-s001.zip › ijms-670590-SI/Table S6.docx]

**Table S6.** Results of pairwise comparisons of dietary treatments across the different *Drosophila* genetic backgrounds.

| Pairs | df | Sums of squares | F.Model | R^2^ | p.value | p.adjusted |
| --- | --- | --- | --- | --- | --- | --- |
| Habanero vs Serrano | 1 | 0.0533 | 2.8565 | 0.2221 | 0.069 | 0.414 |
| Habanero vs Bell | 1 | 0.011 | 0.6215 | 0.0585 | 0.538 | 1 |
| Habanero vs Control | 1 | 0.0163 | 0.9064 | 0.0831 | 0.51 | 1 |
| Serrano vs Bell | 1 | 0.0248 | 2.2403 | 0.1830 | 0.12 | 0.72 |
| Serrano vs Control | 1 | 0.0902 | 7.9848 | 0.4439 | 0.007 | 0.042 |
| Bell vs Control | 1 | 0.0228 | 2.1848 | 0.1793 | 0.104 | 0.624 |
