# Supplementary figures and images for: Effect of Pepper-Containing Diets on the Diversity and Composition of Gut Microbiome of Drosophila melanogaster
Source: Int J Mol Sci. 2020 Jan 31;21(3):945. doi: 10.3390/ijms21030945 (PMC7038135; doi:10.3390/ijms21030945)

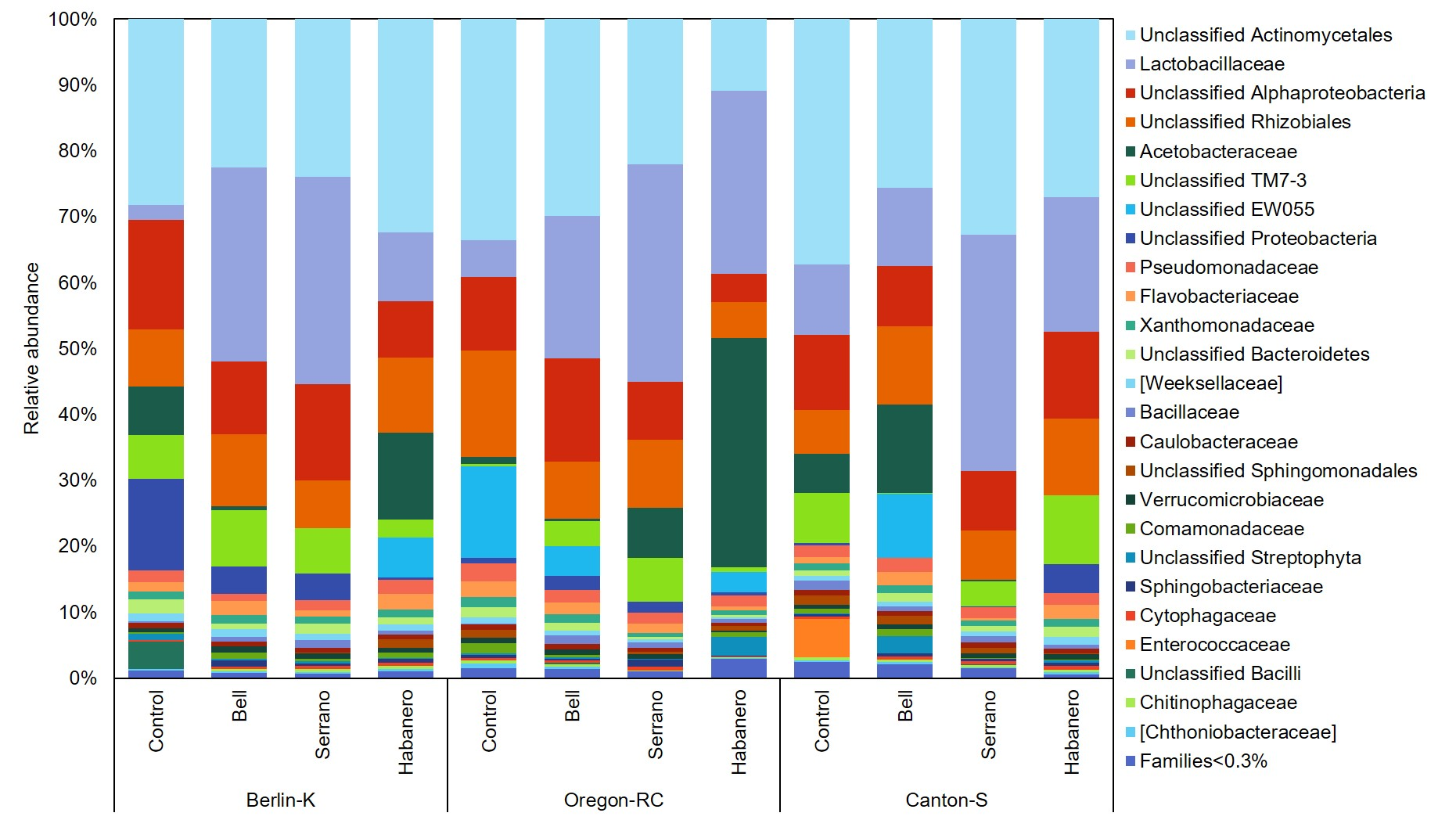

Supplement: Supplementary file 1 [file ijms-21-00945-s001.zip › ijms-670590-SI/Fig.S2.tif]

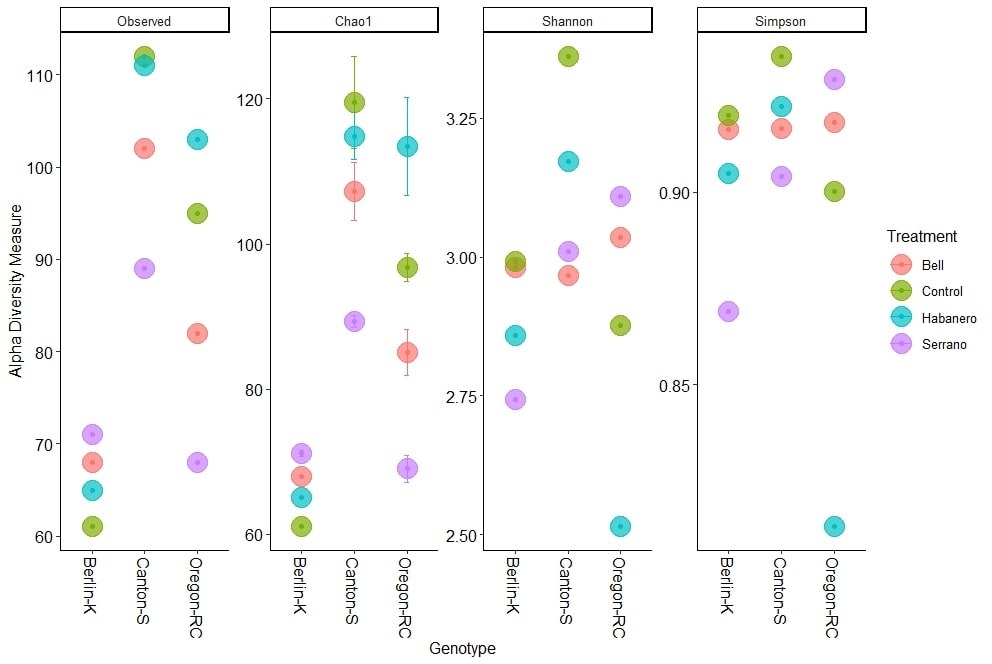

Supplement: Supplementary file 1 [file ijms-21-00945-s001.zip › ijms-670590-SI/Figure S1.jpg]

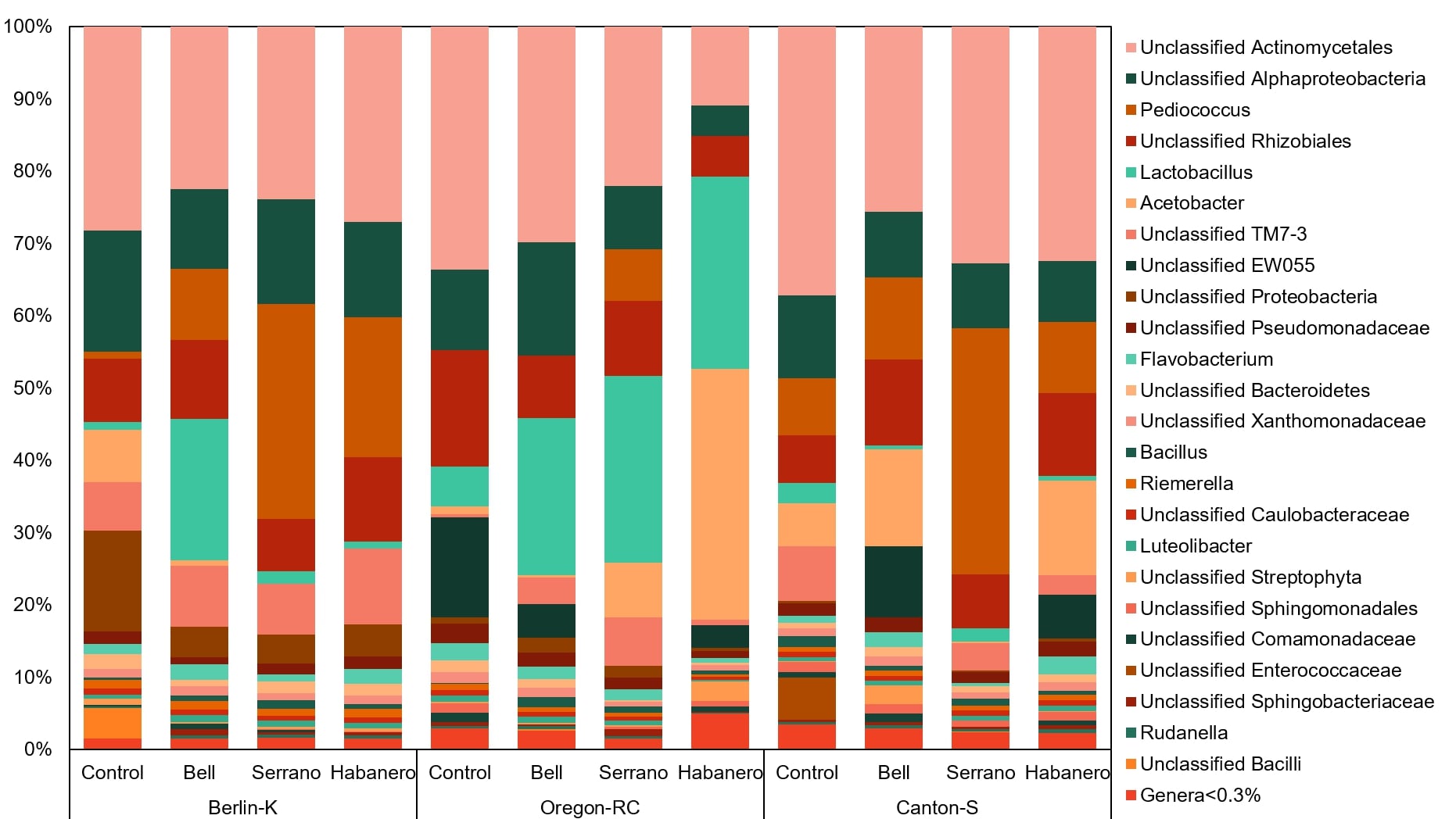

Supplement: Supplementary file 1 [file ijms-21-00945-s001.zip › ijms-670590-SI/Figure S4.jpg]
